# Folding into shape

**DOI:** 10.7554/eLife.93122

**Published:** 2023-11-09

**Authors:** Héloïse de Vareilles

**Affiliations:** 1 Department of Psychiatry, University of Cambridge Cambridge United Kingdom

**Keywords:** folding, cerebellum, evolution, histology, mammals, phylogenetic comparative methods, Other

## Abstract

A new computational tool provides insights into the structure of the cerebellum in mammals.

**Related research article** Heuer K, Traut N, de Sousa AA, Valk SL, Clavel J, Toro R. 2023. Diversity and evolution of cerebellar folding in mammals. *eLife* **12**:e85907. doi: 10.7554/eLife.85907.

During development, the brains of most mammals (including humans) will fold into a unique pattern of grooves and ridges. Understanding how these wrinkles emerge can provide important insights into how the brain works. Most research in this area has focused on the structure of the cerebrum, the two large lobes that make up most of the brain. However, much less is known about the structure of the cerebellum – the ‘little brain’ that sits beneath the cerebrum.

The anatomical properties of the cerebrum – such as the number of folds it contains, its thickness and surface area – have been shown to vary between mammalian species and to influence cognitive processes in humans (Mota and Herculano-Houzel, 2015, Cachia et al., 2021). Unlike the cerebrum, which is folded in some mammals (such as humans) but flat in others (such as mice), the cerebellum always has a wrinkled appearance. The structure of the cerebellum is also more uniform across different species (including some invertebrates), and this has led to the assumption that its folds have less of an influence on cognition than the folds of the cerebrum. However, recent work suggests that the cerebellum may have a bigger role in cognition than previously thought (Buckner, 2013).

In 2020, Ken Ashwell of the University of New South Wales compared the volumes of the cerebellum and cerebrum in monotremes, marsupials and eutherian mammals (Ashwell, 2020). However, the study did not dive into the detailed anatomy of the cerebellum, as its small size and highly folded configuration are difficult to examine using conventional methods. Now, in eLife, Roberto Toro from the Institut Pasteur and colleagues – including Katja Heuer as first author – report a new approach for investigating the shape of the cerebellum in mammals (Heuer et al., 2023).

The team developed a computational method that can measure the surface area of the cerebellum, the shape of its individual folds, and the thickness of its most superficial layer (Figure 1A). The approach, which is freely available, was validated by ensuring that it could produce results that fitted with previously reported data. It was then applied to histological slices extracted from the cerebellums of 56 different mammals, including slices examined in the Ashwell study (Figure 1B).

**Figure 1.**
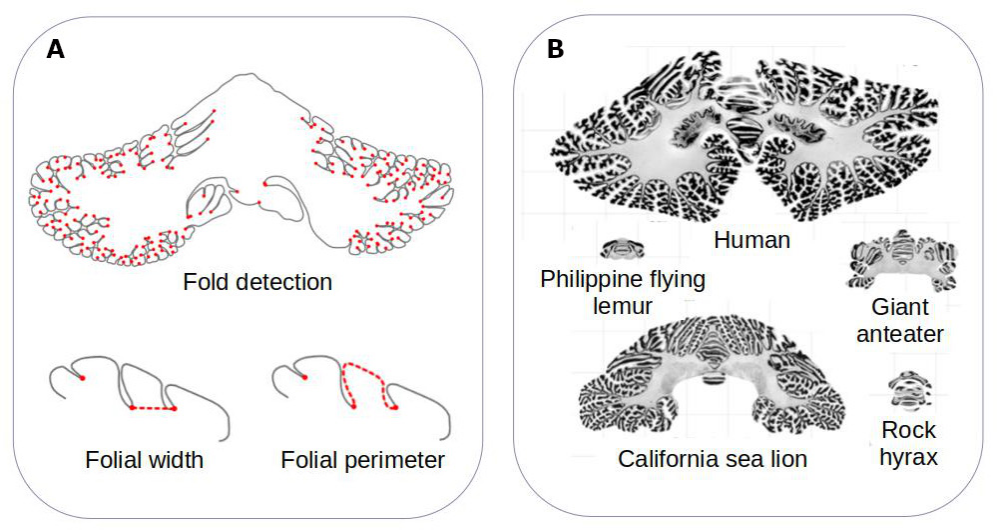
Comparing the structure of the cerebellum in mammalian species. (**A**) Heuer et al. created a computational model that can automatically identify grooves in the cerebellum and measure various metrics (shown in red), including the width (bottom left) and perimeter (bottom right) of individual folds. (**B**) The model was applied to histological slices extracted from the cerebellums of 56 mammals. The results of this analysis are shown for five species (which are displayed on the same scale). Heuer et al. estimated that most mammals are descended from a species that had a cerebellum similar to that of the rock hyrax (bottom right).

Image credit: Adapted from Figure 1 and Figure 3 of the manuscript by Heuer et al., 2023

Heuer et al. – who are based at various institutes in France, the United Kingdom and Germany – found that the different folding metrics detected by their model could be split in to two groups: ones that varied a lot between species (such as surface area), and ones that did not vary as much (such as the width of individual folds). While body size varied by 11 orders of magnitude within the sample, the surface area of the cerebellum varied much less (by around 2.5 orders of magnitude), and the width between two folds only varied by 0.5 orders of magnitude. This suggests that larger mammals have larger cerebellums, but the size of their folds are relatively thin in comparison.

The folding metrics measured also scaled within species. For instance, bigger cerebellums had a higher number of folds than smaller ones **–** an effect which has also been observed in the cerebrum (Germanaud et al., 2012). Species with larger cerebrums also appeared to have relatively smaller cerebellums.

Heuer et al. then set out to find which computational model of evolution was able to recreate the anatomies of the cerebellums found in the different mammalian species. They found that a stabilising model – that is, when animals that are straying away from their common ancestor randomly evolve back to these initial properties – worked best. Using this model, Heuer et al. estimated that the organism most mammals descended from probably had a cerebellum similar to the one found in an animal known as the rock hyrax (Figure 1B).

These observations, along with other results reported by Heuer et al., provide food for thought about the mechanics of brain folding. Although the composition of cells within the cerebellum differ from those in the cerebrum, Heuer et al. propose that these parts of the brain fold in the same way, with the most superficial layer expanding more than the layer immediately below. This process, along with neurons in the cerebellum developing in a certain way, may lead to specific patterns of grooves and ridges forming in the cerebellums of different species. In the future, the new tool created by Heuer et al. could be used to see if these observations occur in the cerebellums of other species (including non-mammals), and to look in to the ‘folds within folds’ that appear in larger cerebellums in more detail.
